# Supplementary material for: Dysregulation of Epigenetic Mechanisms of Gene Expression in the Pathologies of Hyperhomocysteinemia
Source: Int J Mol Sci. 2019 Jun 27;20(13):3140. doi: 10.3390/ijms20133140 (PMC6651274; doi:10.3390/ijms20133140)
Supplement: Supplementary file 1 [file ijms-20-03140-s001.pdf]

Table S1. Effects of HHcy on gene expression in vitro

Cell line	Treatment	Upregulated pathways	Downregulated pathways	Reference
**HUVEC**	10, 100, or 1000 μM D, L-Hcy 24 h	Transmembrane transport, mTOR signaling, Transcription activation, Immunity.	Transmembrane transport, Transcription activation, Immunity, Cell adhesion, Neurogenesis/signal transduction, N-glycan processing, Cytoskeleton assembly, Endoplasmic reticulum quality control.	[1]
**HUVEC**	10, 100, or 1000 μM L-HTL 24 h	Chromatin modification/assembly, Histone Methylation/transcription, Folate and one-carbon metabolism, Lipid transport, Lipid metabolism, Cell adhesion, Angiogenesis, Proteolysis, Endothelial cell cycle, Cytoskeleton function, Wnt signaling, mTOR signaling, Endocytotic signaling, Apoptotic signaling, Phospholipid metabolism, Blood clotting, Oxygen transport, Detoxification, Extracellular matrix organization, Purine biosynthesis, Nucleotide metabolism, Endoplasmic reticulum stress, Organic anion transport, Glucose transport, Lysosome function, Immunity.	Chromatin modification/transcription regulation, Nucleosome assembly, One-carbon metabolism, Lipid metabolism/transport, Angiogenesis, Blood coagulation, Cell adhesion, Immunity/cell adhesion, Energy metabolism/glycolysis, Cytokine signaling, Signaling, Glycolipid biosynthesis, Cell morphology, Protein ubiquitination, Protein deubiquitination, Transmembrane transport, Cytoskeleton assembly, Extracellular matrix organization, Endoplasmic reticulum Quality control, N-glycan processing.	[1]
**HUVEC**	10 or 40 μM *N*-Hcy-FBS, 24 h	Regulation of cell morphology, Endoplasmic reticulum quality control, Protein biosynthesis, Angiogenesis, Protein folding, mRNA splicing, Transcription regulation, Detoxification,	Phagocytosis, Proteolysis, Protein biosynthesis, Peptide hormone processing, Transcription regulation, RNA processing or transport, Signal transduction, Cytoskeleton assembly, Energy metabolism,	[1]

		Membrane transport,<br>Lipid metabolism	Leukotriene metabolism,<br>Host defense system,<br>Sulfation,<br>N-glycan processing	
**Human hepatocytes in primary short-term cultures**	2 mM D,L-Hcy, 48 h	Pyruvate metabolism,<br>Oxidative stress response,<br>Gluconeogenesis,<br>Amino acid biosynthesis,<br>Lipid metabolism	Ribosome assembly	[2]
**Cultured human skin fibroblasts Control and *cblC* mutant fibroblasts**	NA		Glutathione metabolism,<br>Apoptosis	[3]
**VSMCs from thoracic aorta of adult Wistar rats**	0.1–1 mM Hcy, 48 h;<br>0.5 mM Hcy, 0–72 h	Glucose metabolism<br>Cytoskeletal protein: Vimentin,<br>Calreticulin,<br>Similar to WDR1 protein	Cytoskeletal protein: Lamin C,<br>LIM and SH3 protein 1	[3]

Table S2. Effects of HHcy on gene expression in vivo

Organism, treatment & tissue	Upregulated pathways	Downregulated pathways	Reference
***Tg-I278T Cbs**$^{-/-}$* **mice Liver**	Cytokine-cytokine receptor interaction,<br>Metabolism of xenobiotics by cytochrome P450,<br>Focal adhesion,<br>Arachidonic acid metabolism,<br>Fructose and mannose metabolism,<br>Pyrimidine metabolism,<br>TGF-beta signaling,<br>Chronic myeloid leukemia,<br>Glycolysis/ Gluconeogenesis,<br>Glycine, serine and threonine metabolism,<br>Tyrosine metabolism,<br>mTOR signaling,<br>Pentose phosphate,<br>Histidine metabolism,<br>Glycan structures – degradation,<br>C21-Steroid hormone metabolism	PPAR signaling,<br>Neuroactive ligand-receptor interaction,<br>MAPK signaling,<br>Adipocytokine signaling,<br>Androgen and estrogen metabolism,<br>Phosphatidylinositol signaling,<br>Nicotinate and nicotinamide metabolism,<br>Methionine metabolism,<br>C21-Steroid hormone metabolism,<br>Benzoate degradation via CoA ligation,<br>Valine, leucine, and isoleucine degradation,	[4]
***Tg-hCBS Cbs**$^{-/-}$* **mice Liver**	Cytokine-cytokine receptor interaction,<br>Calcium signaling,	MAPK signaling,<br>PPAR signaling,<br>Insulin signaling,	[4]

	Focal adhesion,<br>Leukocyte transendothelial migration,<br>Huntington's disease,<br>Androgen and estrogen metabolism,<br>C21-Steroid hormone metabolism	Adipocytokine signaling,<br>Pyruvate metabolism,<br>Type II diabetes mellitus,<br>Glycerolipid metabolism,<br>Fructose and mannose metabolism,<br>Fatty acid biosynthesis/metabolism	
***Cbs*$^{+/-}$ mice**<br>**Both genders**<br>**Liver**	Urea cycle,<br>Met metabolism,<br>Arg and Pro metabolism,<br>Phe, Tyr and Trp biosynthesis,<br>Selenoamino acid metabolism,<br>Glutamate metabolism,<br>Proteolysis,<br>Lipid transport,<br>Transcription		[5]
**WT mice ± 0.5% Met in drinking water**<br>**Both genders**<br>**Liver**	Urea cycle,<br>Met metabolism,<br>Arg and Pro metabolism,<br>Glycolysis/gluconeogenesis,<br>Amino acid metabolism,<br>Butanoate metabolism,<br>Pyruvate metabolism,<br>Propanoate metabolism,<br>Fatty acid metabolism,<br>Bile acid metabolism,<br>Ascorbate metabolism		[5]
***Cbs*$^{+/-}$ mice ± 0.5% Met in drinking water**<br>**Both genders**<br>**Liver**	Urea cycle,<br>Met metabolism,<br>Arg and Pro metabolism,<br>Glycolysis/gluconeogenesis,<br>Amino acid metabolism,<br>Butanoate metabolism,<br>Pyruvate metabolism,<br>Propanoate metabolism,<br>Fatty acid metabolism,<br>Bile acid metabolism,<br>Ascorbate metabolism		[5]
***Blmh*$^{-/-}$ C57BL/6J mice**<br>**Liver**	Nitric oxide generation,<br>Xenobiotic detoxification	Lipoprotein metabolism,<br>Energy metabolism,<br>Methylglyoxal detoxification,<br>Antioxidant defense	[6]
**C57BL/6J**<br>**1% Met in drinking water (8 weeks)**<br>**Liver**	Antigen processing,<br>Energy metabolism,<br>Iron metabolism and homeostasis,<br>Oxidative stress response,<br>Catechol metabolism	Lipoprotein metabolism,<br>Nitric oxide generation,<br>Methylglyoxal detoxification,<br>Xenobiotic detoxification	[6]

Blmh$^{-/-}$ **C57BL/6J mice 1% Met in drinking water (8 weeks) Liver**	Oxidative stress response, Nitric oxide generation, Xenobiotic detoxification	Lipoprotein metabolism, Antigen processing, Energy metabolism, Iron metabolism/homeostasis, Catechol metabolism, Methylglyoxal detoxification	[6]
**Female *Blmh***$^{-/-}$ **C57BL/6J mice Kidney**	Carbohydrate metabolism, Oxidative stress response	Lipoprotein metabolism, Amino acid and protein metabolism, Energy metabolism, Carbohydrate metabolism	[7]
**Female C57BL/6J mice 1% Met in drinking water (8 weeks) Kidney**	Amino acid and protein metabolism, Carbohydrate metabolism, Oxidative stress response	Lipoprotein metabolism, Energy metabolism, Carbohydrate metabolism	[7]
**Female *Blmh***$^{-/-}$ **C57BL/6J mice 1% Met in drinking water (8 weeks) Kidney**	Amino acid and protein metabolism, Energy metabolism, Carbohydrate metabolism, Oxidative stress response	Lipoprotein metabolism, Carbohydrate metabolism	[7]
**Female *Blmh***$^{-/-}$ **C57BL/6J mice Brain**	Brain-specific, Antioxidant defense, Energy metabolism, Cell cycle proteins, Cytoskeleton assembly, Iron metabolism, Other proteins	Antioxidant defense, Energy metabolism	[8]
**Female C57BL/6J mice 1% Met in drinking water (8 weeks) Brain**	Cytoskeleton assembly	Brain-specific, Antioxidant defense, Energy metabolism, Cell cycle proteins, Iron metabolism, Other proteins	[8]
**Female *Blmh***$^{-/-}$ **C57BL/6J mice 1% Met in drinking water (8 weeks) Brain**	Brain-specific, Antioxidant defense, Energy metabolism, Cell cycle proteins, Iron metabolism	Brain-specific, Cytoskeleton assembly, Cell cycle proteins	[8]
Pon1$^{-/-}$ **C57BL/6J mice Liver**	Lipoprotein metabolism, Energy metabolism, Iron metabolism, Oxidative stress response, Catechol metabolism, Nitric oxide generation	Lipoprotein metabolism	[9]
**C57BL/6J 1% Met in drinking water (8 weeks) Liver**	Energy metabolism, Iron metabolism, Oxidative stress response, Catechol metabolism, Nitric oxide generation	Lipoprotein metabolism, Energy metabolism	[9]
Pon1$^{-/-}$ **C57BL/6J mice**	Iron metabolism,	Lipoprotein metabolism,	[9]

**1% Met in drinking water for 8 weeks Liver**	Oxidative stress response, Nitric oxide generation	Energy metabolism, Catechol metabolism	
***Pon1*$^{-/-}$ C57BL/6J mice Kidney**	Oxidative stress response	Lipoprotein metabolism, Protein metabolism, Energy metabolism, Carbohydrate metabolism	[10]
**C57BL/6J mice, 1% Met in drinking water (8 weeks) Kidney**	Carbohydrate metabolism, Oxidative stress response	Lipoprotein metabolism, Protein metabolism, Energy metabolism, Carbohydrate metabolism	[10]
***Pon1*$^{-/-}$ C57BL/6J mice, 1% Met in drinking water (8 weeks) Kidney**	Carbohydrate metabolism, Oxidative stress response	Protein metabolism	[10]
***Pon1*$^{-/-}$ C57BL/6J mice Brain**	Cytoskeleton assembly	Brain-specific, Antioxidant defense, Energy metabolism, Cell cycle proteins, Cytoskeleton assembly	[11]
**C57BL/6J mice, 1% Met in drinking water (8 weeks) Brain**	Cytoskeleton assembly	Brain-specific, Antioxidant defense, Energy metabolism, Cell cycle proteins	[11]
***Pon1*$^{-/-}$ C57BL/6J mice, 1% Met in drinking water (8 weeks) Brain**	Brain-specific, Antioxidant defense, Energy metabolism, Cell cycle proteins, Cytoskeleton assembly		[11]
**21-day-old Wistar rat pups from mothers fed one month before mating with control or methyl donor-deficient diet**	Energy production, Lipid and lipoprotein metabolism, Lipid transport, Response to ER stress, Oxidative stress response, Cell structure	Energy production, Lipid and lipoprotein metabolism, Lipid transport, Response to ER stress, Oxidative stress response, Cell structure	[12]
**C57BL/6 mice, high-Met diet, hippocampus**	Regulation of cell shape, Intracellular protein, transport mRNA processing, Glycolysis, Vesicle-mediated transport, RNA splicing, Angiogenesis	Cell adhesion, G1/S transition of mitotic cell cycle, Calcium ion transport, Protein amino acid autophosphorylation, Regulation of cell shape, Embryonic development, Cortical actin cytoskeleton organization and biogenesis, Cytokinesis, Muscle contraction, Neuron migration, Actin filament-based movement,	[13]

		Actin cytoskeleton organization and biogenesis, Lactation, Cell motility, Actin filament bundle formation, Brain development, Calcium ion homeostasis	
***CBS$^{-/-}$* patients plasma**	Immune response, Acute-phase response blood coagulation	Acute-phase response, Vitamin transport, Complement/coagulation cascades, Fat digestion/absorption, Cholesterol transport, Antioxidant activity, Blood coagulation, Negative regulation of amyloid-beta formation, Cellular iron ion homeostasis, Amine metabolic process, Blood coagulation/inflammatory response, Retinol metabolic process, thyroid hormone transport, Vitamin D metabolic process	[14]

References

1. Gurda, D., Handschuh, L., Kotkowiak, W., and Jakubowski, H. (2015) Homocysteine thiolactone and N-homocysteinylated protein induce pro-atherogenic changes in gene expression in human vascular endothelial cells. *Amino acids* **47**, 1319-1339
2. Selicharová, I., Kořínek, M., Demianová, Z., Chrudinová, M., Mládková, J., and Jiráček, J. (2013) Effects of hyperhomocysteinemia and betaine-homocysteine S-methyltransferase inhibition on hepatocyte metabolites and the proteome. *Biochim Biophys Acta* **1834**, 1596-1606
3. Liu, X., Shen, J., Zhan, R., Wang, X., Zhang, Z., Leng, X., Yang, Z., and Qian, L. (2009) Proteomic analysis of homocysteine induced proliferation of cultured neonatal rat vascular smooth muscle cells. *Biochim Biophys Acta* **1794**, 177-184
4. Gupta, S., Kuhnisch, J., Mustafa, A., Lhotak, S., Schlachterman, A., Slifker, M. J., Klein-Szanto, A., High, K. A., Austin, R. C., and Kruger, W. D. (2009) Mouse models of cystathionine beta-synthase deficiency reveal significant threshold effects of hyperhomocysteinemia. *FASEB journal : official publication of the Federation of American Societies for Experimental Biology* **23**, 883-893
5. DiBello, P. M., Dayal, S., Kaveti, S., Zhang, D., Kinter, M., Lentz, S. R., and Jacobsen, D. W. (2010) The nutrigenetics of hyperhomocysteinemia: quantitative proteomics reveals differences in the methionine cycle enzymes of gene-induced versus diet-induced hyperhomocysteinemia. *Mol Cell Proteomics* **9**, 471-485
6. Suszynska-Zajczyk, J., Wroblewski, J., Utyro, O., Luczak, M., Marczak, L., and Jakubowski, H. (2014) Bleomycin hydrolase and hyperhomocysteinemia modulate the expression of mouse proteins involved in liver homeostasis. *Amino acids* **46**, 1471-1480
7. Suszynska-Zajczyk, J., Utyro, O., and Jakubowski, H. (2014) Methionine-induced hyperhomocysteinemia and bleomycin hydrolase deficiency alter the expression of mouse kidney proteins involved in renal disease. *Molecular genetics and metabolism* **112**, 339-346
8. Suszynska-Zajczyk, J., Luczak, M., Marczak, L., and Jakubowski, H. (2014) Hyperhomocysteinemia and bleomycin hydrolase modulate the expression of mouse brain proteins involved in neurodegeneration. *Journal of Alzheimer's disease : JAD* **40**, 713-726

9. Suszynska-Zajczyk, J., and Jakubowski, H. (2014) Paraoxonase 1 and dietary hyperhomocysteinemia modulate the expression of mouse proteins involved in liver homeostasis. *Acta biochimica Polonica* **61**, 815-823
10. Suszynska-Zajczyk, J., Sikora, M., and Jakubowski, H. (2014) Paraoxonase 1 deficiency and hyperhomocysteinemia alter the expression of mouse kidney proteins involved in renal disease. *Molecular genetics and metabolism* **113**, 200-206
11. Suszynska-Zajczyk, J., Luczak, M., Marczak, L., and Jakubowski, H. (2014) Inactivation of the paraoxonase 1 gene affects the expression of mouse brain proteins involved in neurodegeneration. *Journal of Alzheimer's disease : JAD* **42**, 247-260
12. Martinez, E., Gérard, N., Garcia, M. M., Mazur, A., Guéant-Rodriguez, R. M., Comte, B., Guéant, J. L., and Brachet, P. (2013) Myocardium proteome remodelling after nutritional deprivation of methyl donors. *J Nutr Biochem* **24**, 1241-1250
13. Fang, M., Wang, J., Yan, H., Zhao, Y. X., and Liu, X. Y. (2014) A proteomics study of hyperhomocysteinemia injury of the hippocampal neurons using iTRAQ. *Mol Med Rep* **10**, 2511-2516
14. Sikora, M., Lewandowska, I., Kupc, M., Kubalska, J., Graban, A., Marczak, Ł., Kaźmierski, R., and Jakubowski, H. (2019) Serum proteom alterations in human cystathionine β-synthase deficiency and ischemic stroke subtypes. *Int J Mol Sci* **20**, 3096
